# Temperature- and pressure-dependent stopped-flow kinetic studies of jack bean urease. Implications for the catalytic mechanism

**DOI:** 10.1007/s00775-012-0926-8

**Published:** 2012-08-14

**Authors:** Barbara Krajewska, Rudi van Eldik, Małgorzata Brindell

**Affiliations:** 1 Faculty of Chemistry, Jagiellonian University, Ingardena 3, 30-060 Kraków, Poland; 2 Department of Chemistry and Pharmacy, Friedrich Alexander University Erlangen-Nürnberg, Egerlandstrasse 1, 91058 Erlangen, Germany

**Keywords:** Urease, Catalytic mechanism, Temperature and pressure dependence, Thermodynamic and activation parameters, Stopped flow

## Abstract

**Abstract:**

Urease, a Ni-containing metalloenzyme, features an activity that has profound medical and agricultural implications. The mechanism of this activity, however, has not been as yet thoroughly established. Accordingly, to improve its understanding, in this study we analyzed the steady-state kinetic parameters of the enzyme (jack bean), *K*_M_ and *k*_cat_, measured at different temperatures and pressures. Such an analysis is useful as it provides information on the molecular nature of the intermediate and transition states of the catalytic reaction. We measured the parameters in a noninteracting buffer using a stopped-flow technique in the temperature range 15–35 °C and in the pressure range 5–132 MPa, the pressure-dependent measurements being the first of their kind performed for urease. While temperature enhanced the activity of urease, pressure inhibited the enzyme; the inhibition was biphasic. Analyzing *K*_M_ provided the characteristics of the formation of the ES complex, and analyzing *k*_cat_, the characteristics of the activation of ES. From the temperature-dependent measurements, the energetic parameters were derived, i.e. thermodynamic Δ*H* ^o^ and Δ*S* ^o^ for ES formation, and kinetic Δ*H* ^*≠*^ and Δ*S* ^*≠*^ for ES activation, while from the pressure-dependent measurements, the binding Δ*V*_b_ and activation $\Updelta V_{\rm cat}^{ \ne }$ volumes were determined. The thermodynamic and activation parameters obtained are discussed in terms of the current proposals for the mechanism of the urease reaction, and they are found to support the mechanism proposed by Benini et al. (*Structure* 7:205–216; 1999), in which the Ni–Ni bridging hydroxide—not the terminal hydroxide—is the nucleophile in the catalytic reaction.

**Graphical abstract:**

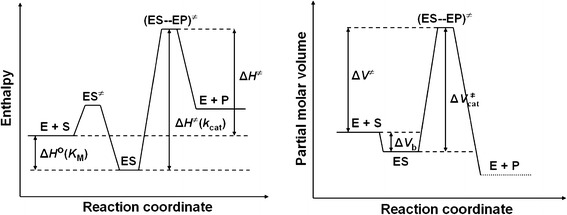

## Introduction

**Scheme 1:**
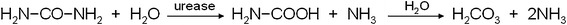

Ureases (urea amidohydrolases, EC 3.5.1.5) are high molecular weight, multisubunit, Ni-containing metalloenzymes [1] that are found in numerous bacteria, plants, fungi, algae, and some invertebrates, as well as in soils as a soil enzyme [2, 3]. Bacterial ureases differ from plant and fungal ones, typically homohexameric, in that they are composed of heteromeric subunits. Yet, irrespective of their origin, ureases fulfill one catalytic function: to hydrolyze urea [2, 3]. The immediate products of this enzymatic reaction are NH_3_ and carbamate; however, the observed products are NH_3_ and H_2_CO_3_, due to the spontaneous hydrolysis of carbamate (Scheme 1). These reactions cause a significant increase in pH.

Notwithstanding that urease was the first enzyme ever crystallized (1926) [4] and extensively studied over the years, its catalytic mechanism still remains disputable [5, 6]. The elucidation of this mechanism is of importance for counteracting undesirable effects generated by the enzyme. These include the product NH_3_ and an increase in pH, both capable of causing deleterious complications, notably in medicine and agriculture [2, 3]. In medicine, bacterial ureases may act as virulence factors that give rise to pathological conditions, such as peptic ulcer disease, gastric cancer, and hepatic coma resulting from infection of the gastrointestinal tracts (primarily with *Helicobacter pylori*), as well as kidney stone formation and pyelonephritis, resulting from infection of the urinary tracts (chiefly with *Proteus mirabilis* and *Ureaplasma urealyticum*). In agriculture, by contrast, if the hydrolysis of fertilizer urea by soil urease is too rapid, it can lead to the unproductive volatilization of nitrogen, and may cause ammonia toxicity and alkaline-induced plant damage. Various strategies have been utilized to combat these complications. One of them is to disable urease through the use of inhibitors [7–9].

Several classes of compounds are known to inhibit ureases [3], including amides and esters of phosphoric acid [5, 10], thiols [11], hydroxamic acids [12], phosphinic and thiophosphinic acids [13], boric and boronic acids [14, 15], phosphate [16], heavy metal ions [17, 18], bismuth compounds [19], quinones [20, 21], and to a lesser extent H_2_O_2_ [22], as well as L-ascorbic and dehydroascorbic acid in the presence of Fe^3+^ ions [23]. Due to their toxicity, however, only few of the compounds may classify as medicinal and agricultural agents.

Thus, further to theoretical knowledge of urease biochemistry, a thorough understanding of the catalytic mechanism of the enzyme is indispensable for devising an effective, dependable and safe manner of controlling its activity.

### Active site of urease

**Scheme 2:**
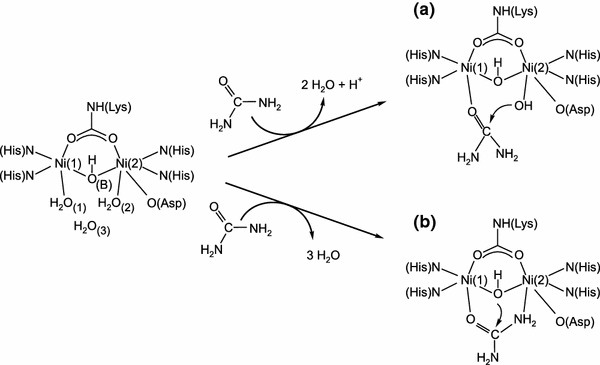

The active site of urease (Scheme 2) contains a binuclear nickel center where nickel(II) ions, separated by a distance of 3.7 Å, are bridged by a carbamylated lysine through its O atoms. Ni(1) is further coordinated by two histidine residues (through their N atoms), and Ni(2) by two histidine residues (also through N atoms) as well as by an aspartic acid residue (through its O atom). The Ni ions are also bridged by a hydroxide ion (WB), which—along with two terminal water molecules (W1 on Ni(1), W2 on Ni(2)) and another water (W3) located towards the opening of the active site—form an H-bonded tetrahedral cluster that fills the active-site cavity. As a result of the above ligations, Ni(1) is pentacoordinate and Ni(2) is hexacoordinate. In addition to the amino acid residues that are directly involved in the architecture of the active site, functional in the urease catalysis are also the residues that compose the mobile flap of the site. Mainly through H-bonding, these residues participate in substrate binding, stabilize the catalytic transition state, and accelerate the reaction.

Remarkably, this active site was found to be almost completely superimposable among ureases from different sources, including bacterial ureases from *Klebsiella aerogenes* [24], *Bacillus pasteurii* [5], and *Helicobacter pylori* [25], and the plant urease from *Canavalia ensiformis* (jack bean) [26]. This is important, in that the conserved active site and consequently the same catalytic mechanism allow the generalization of experimental data to all ureases, independent of their origin.

### Proposed reaction mechanisms for urease-catalyzed urea hydrolysis

The currently proposed mechanisms for the urease-catalyzed hydrolysis of urea are those by Karplus et al. [27] and by Benini et al. [5], developed for *K. aerogenes* and *B. pasteurii* urease, respectively. The mechanisms assume that, in the active site of urease (Scheme 2), urea binds to the more electrophilic Ni(1) ion with the oxygen atom of its carbonyl group, owing to which the carbonyl carbon becomes more electrophilic.

In the mechanism proposed by Karplus et al. [27] (Scheme 2a), urea binds to the active site in a monodentate manner only to Ni(1), with a water molecule retained on Ni(2). Further, acting as a nucleophile, the Ni(2)-coordinated hydroxide attacks the carbonyl atom of the urea molecule to form a tetrahedral intermediate, from which upon the protonation of the leaving amide group, NH_3_ and carbamate are released. The authors argue that the general acid that donates protons to the leaving NH_3_ is His320, located in the mobile flap of the active site. The proposed monodentate urea binding and the suggested catalytic mechanism were supported by molecular dynamics simulations [28] and by an isotope study of the urease-catalyzed hydrolysis of formamide [29]. Nonetheless, several issues associated with this mechanism remain unclear, including the identity of a general base that would deprotonate the Ni(2) water at the optimum pH (~7.5) for activity, and the role of His320, which would need to be protonated at the enzyme’s optimum pH to be able to act as a general acid, even though it has a p*K*_a_ of ~6.5. To explain this, the authors assumed a reverse protonation mechanism; however, the mechanism suffers from having only 0.3 % of the enzyme in the protonation state optimal for the catalysis.

In the other mechanism (Scheme 2b), proposed by Benini et al. [5], a urea molecule replaces the W1–W3 water molecules and, aside from being bound to Ni(1) through its oxygen, it also binds to Ni(2) through the nitrogen of its nonleaving amide group, to form an overall bidentate binding to the metal center. The authors propose that the nucleophile that attacks the carbonyl carbon of urea is the bridging hydroxide, which simultaneously acts as a general acid that delivers protons to the leaving NH_3_ molecules. Upon the attack, a tetrahedral intermediate is formed that breaks down into NH_3_ and carbamate. The authors ascribe a minor role to His323 (His320 according to the residue numbering used for *K. aerogenes*) in stabilizing the positive charge of the leaving N in the transition state. In this mechanism, the issue of reverse protonation is avoided, however, problematic remains the proton transfer between the bridging hydroxide and the distal amide group of urea.

All things considered, the proposed mechanisms of urease catalysis contain a number of controversies that remain to be clarified, primary among them being the urea binding mode and the identities of both the nucleophile and the proton donor.

### Steady-state approach to enzyme kinetics

Though at high concentrations, substrate and product inhibitions are seen, urease typically exhibits Michaelis–Menten kinetics [30] throughout the general scheme [31]:

1 $$ {\text{E}} + {\text{S}}\,\underset{{k_{{ - 1}} }}{\overset{{k_{1} }}{\rightleftarrows}}\,{\text{ES}}\,{{\overset{{k_{2}}}{\rightarrow}}}\,{\text{E}} + {\text{P}} $$

where *k*_1_ and *k*_−1_ are the rate constants for the formation and dissociation of the enzyme–substrate (ES) complex, and *k*_2_ is the rate constant for the breakdown of the ES complex to E and P. If the steady-state approximation is employed, the initial reaction rate is expressed as:

2 $$ v_{0} = \frac{{{\text{d}}P}}{{{\text{d}}t}} = \frac{{k_{1} \;k_{2} \;S}}{{k_{ - 1} + k_{2} + k_{1} S}}\;E = \frac{{v_{\max } S}}{{S + K_{M} }}, $$

where *v*_max_ = *k*_2_ *E* is the maximum reaction rate attained at the saturating substrate concentration. Here, *k*_2_ is the first-order catalytic rate constant *k*_cat_ (hence *v*_max_ = *k*_cat_ *E*) and *E* is the total enzyme concentration. *K*_M_ by contrast, is the Michaelis constant, expressed as:

3 $$ K_{\text{M}} = \frac{{k_{2} + k_{ - 1} }}{{k_{1} }}. $$

When *k*_2_ ≪ *k*_−1_, i.e. the dissociation of ES back to E + S is faster than the formation of P, *K*_M_ becomes the equilibrium constant *K*_D_ for the ES dissociation ES ⇄ E + S:

4 $$ K_{\text{M}} = \frac{{k_{ - 1} }}{{k_{1} }}=K_{\rm D}. $$

However, when *k*_2_ ≫ *k*_−1_, the Michaelis constant becomes:

5 $$ K_{\text{M}} = \frac{{k_{2} }}{{k_{1} }}. $$

### Significance of temperature- and pressure-dependent studies of enzyme kinetics

One pragmatic approach to elucidating enzyme mechanisms is to analyze the steady-state kinetic parameters *K*_M_ and *k*_cat_ for the enzyme, measured at different temperatures [31] and pressures [32]. An analysis of *K*_M_ provides information on how the system changes upon the formation of the ES complex: E + S ⇄ ES, when the binding of the substrate takes place, whereas an analysis of *k*_cat_ provides information on the activation process of the ES complex: ES → (ES–EP)^≠^, when bond reorganization leading to the formation of the products occurs. Using temperature-dependent measurements, the energetic characteristics of the above reaction steps can be obtained: the thermodynamic parameters Δ*H* ^o^ and Δ*S* ^o^ for the formation of ES, and the kinetic parameters Δ*H* ^≠^ and Δ*S* ^≠^ for the formation of the transition state (ES–EP)^≠^. By contrast, using pressure-dependent measurements, information on volume changes associated with the formation of ES, i.e. Δ*V*_b_ (the binding volume), and with the formation of the transition state (ES–EP)^≠^, i.e. $\Updelta V_{\rm cat}^{ \ne }$ (the activation volume), can be derived. Therefore, such an analysis can provide valuable mechanistic information on the molecular nature of the intermediate and transition states of the catalytic reaction.

For ureases, the results of temperature-dependent kinetic analysis are scarce in the literature [30, 33], and intriguingly, disparate in value and sign—likely due to buffer effects. In contrast, pressure-dependent analysis never has been carried out for ureases. Therefore, given its experimental potential, clearly as yet unexploited in the area of urease research, we offer here the results of temperature- and pressure-dependent kinetic analysis of the enzyme (jack bean) activity performed to broaden the understanding of its underlying catalytic mechanism. We studied the kinetics of the reaction using a stopped-flow technique at temperatures between 15 and 35 °C, and at pressures between 5 and 132 MPa—importantly—in a noninteracting biological buffer (HEPES). The obtained thermodynamic and activation parameters are discussed in terms of the current proposals for the mechanism of this reaction.

## Materials and methods

### Materials

Urease (from jack beans, type III, nominal activity 45 U/mg solid), urea (for Molecular Biology), and HEPES buffer (SigmaUltra) were from Sigma (St. Louis, MO, USA). EDTA and phenol red were from POCh (Gliwice, Poland). HEPES buffer 5 mM, pH 6.84, was prepared by diluting a stock 200 mM HEPES solution (pH 7.33) and adding 1 mM EDTA. Ultrapure water (resistivity 18.2 MΩ cm) from a Simplicity 185 water purification system (Millipore, Billerica, MA, USA) was used throughout.

### Urease assay

Given the fact that in the stopped-flow instrument the reaction mixtures are enclosed within the instrument and samples cannot be withdrawn for analysis, for the measurements of the urease reaction rates we chose a pH indicator assay [34] with use of phenol red (p*K*_a_ = 7.9 at 20 °C [34]). The assay makes use of an increase in the pH of the reaction mixture caused by the formation of ammonia during the reaction. The color of phenol red exhibits a gradual transition from yellow to red over the pH range 6.8 to 8.2, thus including the optimum pH of urease activity at 7.0–7.5 [3]. The color transition is followed by the development of absorbance at 560 nm, which was reported to be linear between pH 6.8 and 7.7 [35]. To allow the pH of the reaction mixture to change, we performed the reactions in 5 mM HEPES (p*K*_a_ = 7.55 at 20 °C [36]). Of key importance for the measurements performed in this study was that the p*K*_a_ values of both phenol red and HEPES exhibit little variance with temperature and pressure: for phenol red, Δp*K*_a_/Δ*T* = −0.006/°C [37] and Δp*K*_a_/Δ*p* = −0.0017/MPa [38]; for HEPES buffer, Δp*K*_a_/Δ*T* = −0.014/°C [36] and Δp*K*_a_/Δ*p* = 0.0008/MPa [39].

To choose the correct reaction time, we performed a preliminary experiment which showed that the reaction mixture reached a pH of 7.7 when the reaction was carried out for 5 min at the highest urea concentration of 50 mM. Consequently, the reaction time was set to be up to 200 s at each urea concentration. The initial reaction rates *v*_0_ were calculated from the slope of the linear section of the dependence of the phenol red absorbance at 560 nm on time. To express *v*_0_ in ammonia concentration units (mM NH_3_/s), the change in the absorbance at 560 nm was standardized against the NH_3_ concentration assayed by the colorimetric phenol-hypochlorite method [40], for which the calibration curve was determined independently in 5 mM HEPES at pH 6.84 [41]. The dependence of the absorbance at 560 nm on the NH_3_ concentration was linear up to 1.6 mM NH_3_. By contrast, the concentration of the enzyme urease in the reaction mixture was assessed from the determined activity of the Sigma product, using the activity of the pure enzyme reported to be 6,200 units/mg enzyme on average [6] and the enzyme molecular weight 545.34 kDa [42]. The calculated concentration amounted to 2.305 × 10^−7^ mM, and all subsequent calculations performed in this study refer to the concentration of urease hexamers.

### Stopped-flow measurements of urease kinetics

Temperature-dependent kinetic measurements were performed with a SX20 stopped-flow spectrometer from Applied Photophysics Ltd. (Leatherhead, UK), whereas the pressure-dependent measurements, with a custom-built high-pressure stopped-flow reactor described previously [43]. For the former, the instrument was maintained at atmospheric pressure at five temperatures in the range 15–35 °C, and for the latter, at six pressures in the range 5–132 MPa at 25 °C. Both instruments were thermostated to ±0.1 °C.

To perform the measurements, the following solutions were prepared in 5 mM HEPES buffer at pH 6.84 containing 1 mM EDTA: urease 0.04 mg/mL, phenol red 0.0267 mg/mL, and urea at concentrations between 4 and 200 mM. Next, the phenol red solution was mixed 1:1 with a urea solution of preselected concentration, and the obtained solution was placed in one syringe of the stopped-flow reactor while the other syringe was loaded with the urease solution. The solutions were conditioned in the reactor at a chosen temperature/pressure for 20 min before being mixed 1:1 to initiate the reaction. After mixing, the concentrations in the reaction mixture were: urease 0.02 mg/mL, phenol red 6.675 × 10^−3^ mg/mL, and urea between 1 and 50 mM. The measurements were performed in triplicate and the results were averaged.

The reaction rates *v*_0_ measured at various temperatures and pressures were further used to calculate the steady-state parameters of urease, *K*_M_ and *v*_max_, which was done by the nonlinear least-square fitting of the measured *v*_0_ values to the Michaelis–Menten equation (Eq. 2). The catalytic constant *k*_cat_ was obtained by dividing *v*_max_ by the enzyme concentration.

## Results and discussion

It was assumed in this work that the urease-catalyzed hydrolysis of urea proceeds in two steps according to the Michaelis–Menten mechanism (Eq. 1). The first step is the formation of a stable urease–urea complex, governed by an equilibrium constant equal to the inverse of *K*_D_ (Eq. 4). Importantly, when analyzing the data obtained for this step, we followed the suggestion made in the literature [44]—based on the insignificant variability of *K*_M_ as a function of pH [16, 45]—that urease features *K*_M_ = *K*_D_. By contrast, the second step of the enzymatic reaction involves the activation of the urease–urea complex and its subsequent decomposition into products and free enzyme, and is governed by the catalytic rate constant *k*_cat_. The effects of temperature and pressure on the two governing constants were determined and will be discussed.

**Fig. 1:**
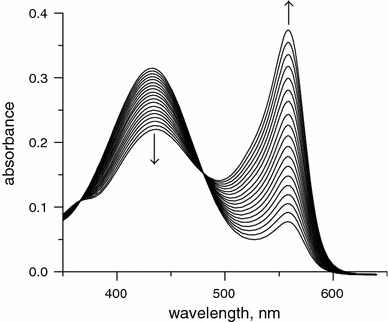
Typical UV–vis spectra of phenol red recorded during a kinetic run for the urease-catalyzed hydrolysis of urea in 5 mM HEPES buffer, pH 6.84, under ambient conditions with 50 mM urea (a PerkinElmer Lambda 35 UV–vis spectrophotometer was used)

In this study, the urease activity was assayed using phenol red. Typical UV–vis spectra for the indicator recorded during a kinetic run performed under ambient conditions with 50 mM urea are presented in Fig. 1. The rise in the absorbance at 560 nm over time was used to obtain *v*_0_.

### Temperature-dependent measurements

The kinetics of urease was studied under atmospheric pressure at temperatures between 15 and 35 °C. Although the optimum temperature for urease activity has been reported to occur in the range 45–65 °C on average [46], temperatures >35 °C were not used in the present experiments to avoid thermal denaturation of the enzyme [47].

**Fig. 2:**
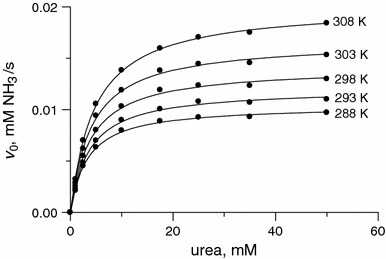
Plots of *v*_0_ versus urea concentration for the urease-catalyzed hydrolysis of urea at temperatures between 15 and 35 °C

The urease saturation curves, *v*_0_ versus *S*, are presented in Fig. 2. They show that the reaction followed Michaelis–Menten kinetics (Eq. 2) at each studied temperature. The kinetic parameters derived from the curves, *K*_M_ and *k*_cat_ (Table 1), were found to be consistent in magnitude with those reported in the literature [44, 45, 48], thus proving that the analytical conditions chosen for the present study were correct. The results obtained show that, as is typically observed for enzymatic reactions, increasing the temperature increased the reaction rate. Accordingly, the *k*_cat_ values grew, increasing twofold between 15 and 35 °C. The *K*_M_ value, on the other hand, in contrast to a previous report of temperature independence [45], showed a slight increase from 3.3 to 4.6 mM, thus indicating a small reduction in the enzyme’s affinity for the substrate at higher temperatures. Apparently, this reduction in affinity can be considered as resulting from the loosening of the active site structure, whose strict architecture is required for the catalysis.

**Table 1:** Kinetic parameters of urease measured at different temperatures and ambient pressure

*T* (K)	*K*_M_ × 10^3^ (M)	*k*_cat_ × 10^−4^ (1/s)
288	3.3 ± 0.2	4.5 ± 0.1
293	3.7 ± 0.2	5.2 ± 0.1
298	3.9 ± 0.2	6.1 ± 0.1
303	4.1 ± 0.2	7.2 ± 0.1
308	4.6 ± 0.1	8.7 ± 0.1

#### Effect of temperature on* K*_M_

Since the binding step E + S ⇄ ES in enzymatic reactions is defined by the equilibrium constant, determining the temperature effects on the constant yields the classical thermodynamic functions Δ*H* ^o^, Δ*S* ^o^, and Δ*G* ^o^ (the standard enthalpy, entropy, and free energy change of the reaction). The equilibrium constant (*K*) and the thermodynamic functions are related as expressed by the following equations:

6 $$ \Updelta G^{\text{o}} = - RT\ln K, $$

7 $$ {\text{and}}\;\Updelta G^{\text{o}} = \Updelta H^{\text{o}} - T\Updelta S^{\text{o}} , $$

8 $$ {\text{hence}}\; - \ln K = \frac{{\Updelta H^{\text{o}} }}{RT} - \frac{{\Updelta S^{\text{o}} }}{R}. $$

**Fig. 3:**
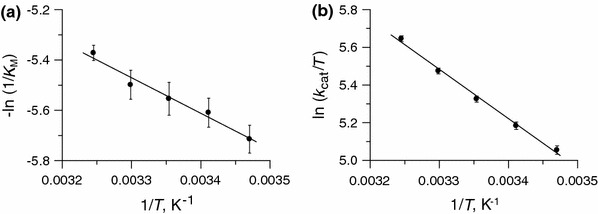
Effect of temperature in the range 15–35 °C on **a** the Michaelis constant *K*_M_ and **b** *k*_cat_/*T* (Eyring plot) for the urease-catalyzed hydrolysis of urea

The Δ*H* ^o^ and Δ*S* ^o^ values can be derived from a linear plot of −ln *K* versus 1/*T* based on Eq. 8, and Δ*G* ^o^ based on Eq. 7. Such a plot was constructed for the dependence of the inverse of the Michaelis constant 1/*K*_M_ for urease on 1/*T* (Fig. 3a). The resulting parameters Δ*H* ^o^, Δ*S* ^o^, and $\Updelta G_{298}^{\text{o}}$ are compiled in Table 2. As shown, the thermodynamic parameters for the formation of the urease–urea complex have favorable values; the reaction is exothermic and spontaneous, and accompanied by a gain in entropy. This entropy gain, however, should be interpreted with caution, since its value is rather small, and it may be a composite of many different contributions that partially cancel each other out. Nevertheless, it may be ascribed, for instance, to the displacement of the ordered water cluster in the active site of urease by a molecule of urea (Scheme 2). Such release of ordered water from proteins has been demonstrated to be responsible for an increase in entropy [49]. Likewise, it may be ascribed to conformational changes in the enzyme that occur when the substrate forms the complex ES, such as a movement of the mobile flap that opens up the active site of urease for urea binding.

**Table 2:** Thermodynamic parameters for the formation of the urease–urea complex in the urease-catalyzed hydrolysis of urea, obtained from temperature-dependent measurements of *K*_M_

Δ*H* ^o^ (kJ/mol)	Δ*S* ^o^ (J/mol K)	$\Updelta G_{298}^{\text{o}}$ (kJ/mol)
−12 ± 1	7 ± 3	−14 ± 2

### Effect of temperature on* k*_cat_

The temperature dependence of *k*_cat_ provides insight into the energetic characteristics of the activation process ES → (ES–EP)^≠^. This process (the catalytic step of the reaction) represents the bond-breaking and/or bond-making step of the reaction, leading to the formation of products. It is characterized by the apparent activation energy *E*_a_ and the changes in the thermodynamic activation functions Δ*H* ^≠^, Δ*S* ^≠^, and Δ*G* ^≠^.

The apparent activation energy *E*_a_ for the catalytic step of the urease reaction was derived from the Arrhenius equation:

9 $$ k_{\text{cat}} = A\;e^{{ - \frac{{E_{a} }}{RT}}} , $$

which gave *E*_a_  = 24 ± 1 kJ/mol. This value corresponds well to the literature values reported for jack bean urease, which are 18–30 kJ/mol on average [46]. To allow a comparison with the literature data, the value of *E*_a_ for 1 mM urea was also calculated. The reaction performed under these conditions has a lower *E*_a_ = 15 ± 1 kJ/mol, which is due to the fact that *v* at a low urea concentration is a composite of *K*_M_ and *k*_cat_, whereas at high urea concentration the *E*_a_ is controlled solely by *k*_cat_. Interestingly, a similar urea concentration effect on *E*_a_ was reported for jack bean urease in THAM buffer [30], but the opposite was observed in phosphate buffer [33], buffer effects being the likely reason for these diverse observations.

The rate constant *k*_cat_ was further analyzed to determine the activation enthalpy and entropy for the catalytic step. To do this, the absolute rate theory was used, where the temperature dependence of the rate constant is expressed by the Eyring–Polanyi equation:

10 $$ \ln \frac{k}{T} = - \frac{{\Updelta H^{ \ne } }}{R}\frac{1}{T} + \ln \frac{{k_{\text{B}} }}{h} + \frac{{\Updelta S^{ \ne } }}{R}, $$

where *R* is the gas constant, *T* is the absolute temperature, *h* is the Planck constant, and *k*_B_ is the Boltzmann constant. The activation enthalpy Δ*H* ^≠^ was extracted from the slope of the Eyring plot, ln (*k*_cat_/*T*) versus 1/*T*, and the activation entropy Δ*S* ^≠^ was derived from the intercept.

**Scheme 3:**
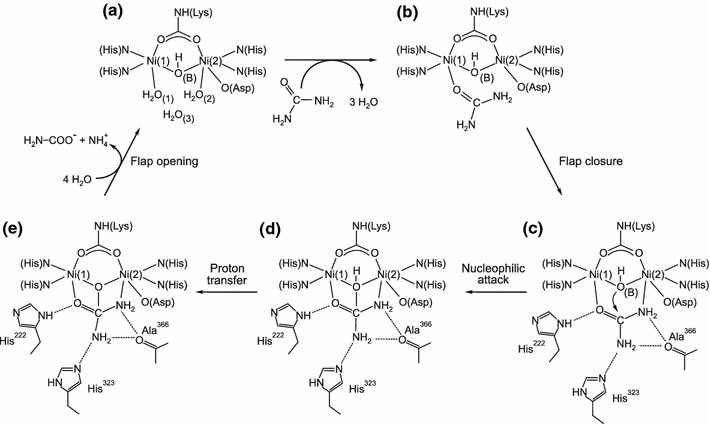

The Eyring plot for the reaction studied here is presented in Fig. 3b, and the resulting values are listed in Table 3. As shown, the reaction is characterized by a positive Δ*H* ^≠^, a negative Δ*S* ^≠^, and a positive Δ*G* ^≠^. The values of both the activation enthalpy Δ*H* ^≠^ (22 kJ/mol) and the entropy Δ*S* ^≠^ (−80 J/K mol) are such that they are unfavorable for lowering the activation free energy Δ*G* ^≠^_298_ (45 kJ/mol) to accelerate the reaction. The large negative value of Δ*S* ^≠^ also indicates that the transition state is more orderly than the ground state of the reactants, thus suggesting that the formation of the transition state does not involve the release of ordered water molecules (Scheme 3).

**Table 3:** Activation parameters for the urease–urea complex in the urease-catalyzed hydrolysis of urea, obtained from temperature-dependent measurements of *k*_cat_

*E*_a_ (kJ/mol)	Δ*H* ^≠^ (kJ/mol)	Δ*S* ^≠^ (J/K mol)	$\Updelta G_{298}^{ \ne }$ (kJ/mol)
24 ± 1	22 ± 1	−80 ± 3	45 ± 2

The activation data obtained in this study (Table 3) diverge in value and sometimes in sign from those reported in previous temperature-dependent studies of urease carried out in THAM [30] and phosphate buffer [33], which is likely due to buffer-dependent effects. Importantly, since HEPES is a noninteracting biological buffer [41, 50], the results collected in this study can be regarded as being independent of buffer effects.

Interestingly, the magnitudes of the *k*_cat_-derived Δ*H* ^≠^ and Δ*S* ^≠^ values for the urease reaction were found to be comparable with those reported for the hydrolysis of *p*-nitrophenyl sulfate catalyzed by arylsulfatase [51]. Therein, it was suggested that such values are supportive of the notion that the activation process consists of an association or interchange rather than of a dissociation. This we find applicable to both of the proposed mechanisms of urea hydrolysis (Scheme 2).

**Fig. 4:**
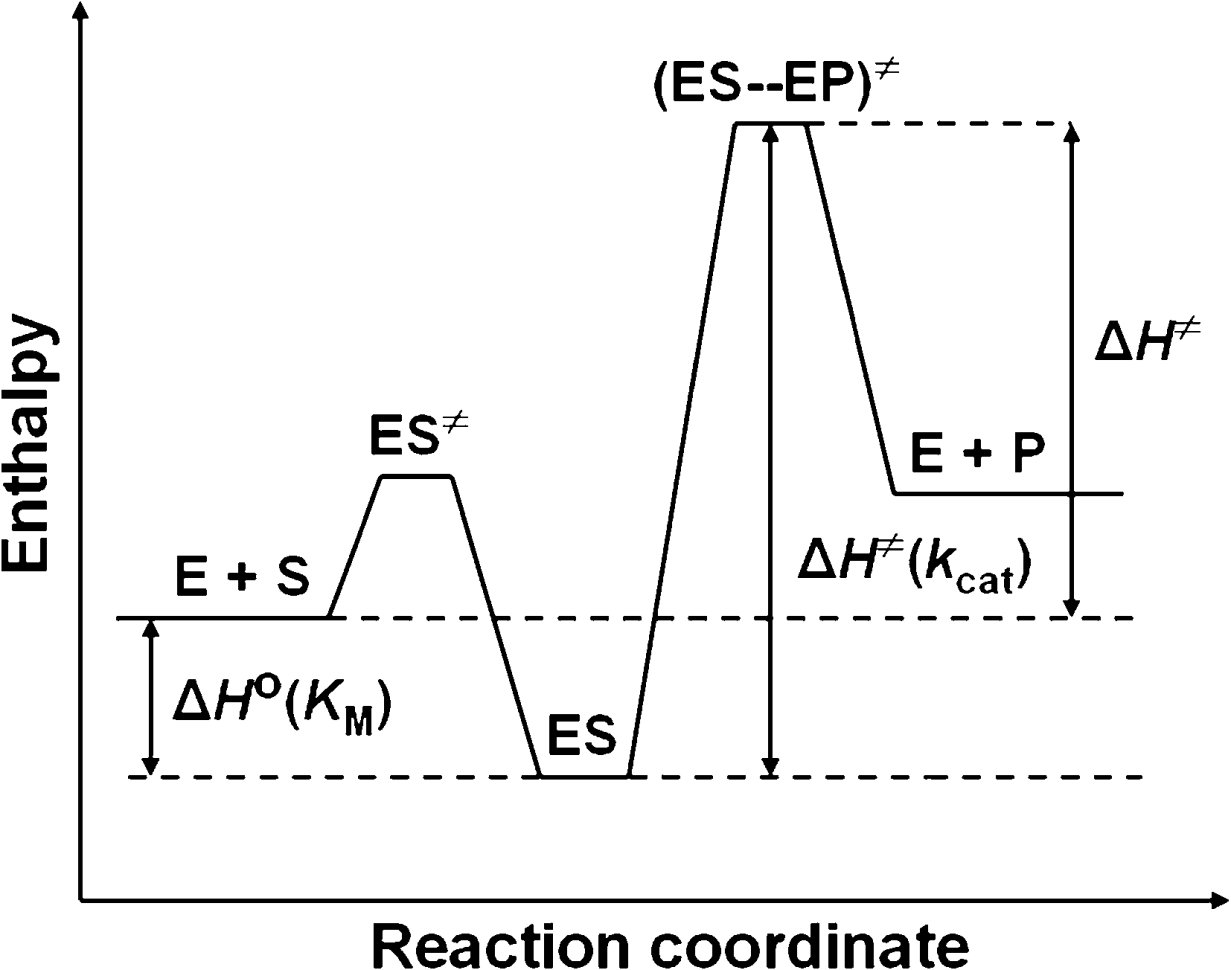
Schematic energy diagram for the urease-catalyzed hydrolysis of urea

Taken together, based on the results obtained in this study and the literature data [31, 52], we propose that the urease-catalyzed hydrolysis of urea follows the energy diagram illustrated in Fig. 4, where Δ*H* ^≠^ = Δ*H* ^≠^(*k*_cat_) − Δ*H* ^o^(*K*_M_).

### Pressure-dependent measurements

Elevated pressure affects enzymes in a complex manner by perturbing the intra- and intermolecular weak, noncovalent interactions that are involved in protein conformation and solvation, and thus responsible for enzyme structure and activity [32, 53–55]. Depending on its magnitude, the pressure may affect protein structures at quaternary, tertiary, and secondary levels with concomitant reductions in their activity, finally leading to denaturation [56]. Thus, there is an upper limit on the pressure that enzymes can endure in the active form. While moderate pressures of 100–200 MPa typically cause the dissociation of oligomeric proteins into subunits, the tertiary protein structures are destroyed by higher pressures of 400–800 MPa, and secondary structures are unaffected by pressures as high as 1,000 MPa. By contrast, the primary structures, which are maintained by covalent bonds, are not pressure sensitive at ordinary temperatures [56, 57]. In addition to obvious structural changes, pressure-induced disturbances of the intra- and intermolecular interactions that occur in enzymes may also cause modifications to the kinetics of enzyme-catalyzed reactions. As a result, measuring enzyme kinetic parameters under increasing pressure provides access to the analysis of the elementary steps of enzyme reactions [32, 53–55].

As enzyme denaturation (subunit dissociation and/or unfolding) may occur under pressure simultaneously with the changes in enzyme kinetics, in order to interpret the experimental results correctly, it is crucial to identify whether the effects observed originated from the former or the latter process [32, 53–55, 58].

Jack bean urease is a large protein made up of six identical subunits, each of molecular mass 90.77 kDa, which are assembled into a hexamer α_6_ [3]. The mass of the hexamer with the 12 nickel ions included is thus 545.34 kDa [42].

In this context, to verify whether our results were an effect of pressure on urease catalysis and not due to the enzyme deactivation by denaturation, that is, weather urease was in the active form during the pressure measurements, we took the following arguments into consideration:

1. In a separate experiment, we checked whether subjecting urease to high pressure for the overall duration of the high-pressure measurements caused a loss of activity through denaturation. In our stopped-flow system, the enzyme solution once set in the apparatus, was subjected to a stepwise increase in pressure (5–132 MPa) until the end of the kinetic measurements performed at each pressure for a series of urea concentrations. It took ca. 2 h to complete the entire experiment. Therefore, to check whether the enzyme was or was not denatured within the duration of the stopped-flow experiment, typical urease samples in 5 mM HEPES buffer were pressurized at 132 MPa for 2 h, and their activities were assayed immediately after depressurization. The results showed that the pressure did not change the activities of the samples. Obviously, this means that the pressure did not bring about an irreversible loss of activity due to denaturation. However, this could also mean that (1) the enzyme dissociates into subunits under pressure but rapidly re-associates without denaturation when depressurized, (2) the enzyme dissociates permanently, but the combined activity of the subunits is the same as that of the hexamer, or (3) there is no dissociation of the enzyme into subunits under applied pressure.
2. Importantly, it was shown in studies on the chemical denaturation of urease to half-units [59] and subunits [60] that the quaternary structure of urease is not required for catalytic activity, and—more importantly–that the subunit is not only the fundamental unit for the quaternary structure of urease but also for its activity.
3. The crystal structures of ureases revealed that the active sites are always located in the α subunits of the enzyme, and are entirely independent [26]. This physical independence of the sites thus supports the notion that the monomeric form of jack bean urease should be active.
4. The fact that the Michaelis constant *K*_M_ of urease shows little variation with pressure (Table 4) apparently confirms that the 3D structure of the enzyme responsible for its activity is practically unperturbed.

**Table 4:** Kinetic parameters of urease measured at different pressures at 25 °C

*p* (MPa)	*K*_M _×_ _10^3^ (M)	*k*_cat _×_ _10^−4^ (1/s)
5	5.0 ± 0.2	6.4 ± 0.1
10	5.3 ± 0.3	6.3 ± 0.1
40	5.5 ± 0.4	6.0 ± 0.1
71	4.3 ± 0.4	4.2 ± 0.1
101	4.7 ± 0.8	3.1 ± 0.1
132	4.1 ± 0.8	2.1 ± 0.1
5. Furthermore, it has been reported that pressure-induced protein denaturation is always accompanied by large negative Δ*V* values [55]; those for the dissociation of oligomeric proteins are typically negative and relatively large between −50 and −200 mL/mol [54], whereas our values clearly do not fall within this range (Table 5).

**Table 5:** Binding and activation volumes in the urease-catalyzed hydrolysis of urea, obtained from pressure-dependent measurements performed at 25 °C

*p* (MPa)	Δ*V*_b_ (*K*_M_) (mL/mol)	$\Updelta V_{\rm cat}^{ \ne }$(*k*_cat_) (mL/mol)	Δ*V* ^≠^ (*K*_M_/*k*_cat_) (mL/mol)
<40	−2 ± 2	5 ± 1	9 ± 4
>40	−2 ± 2	28 ± 1	26 ± 3

Based on the discussion above, we concluded that the pressure applied in this study did not denature urease, and that even if dissociated into subunits the enzyme retained its activity. We used this as the foundation for a further analysis of the effects of pressure on the kinetic parameters of urease.

**Fig. 5:**
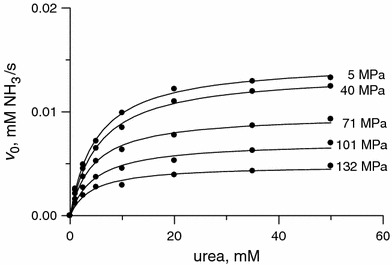
Plots of *v*_0_ versus urea concentration for the urease-catalyzed hydrolysis of urea at pressures between 5 and 132 MPa and 25 °C

The saturation curves for urease obtained in the studied pressure range 5–132 MPa at 25 °C are presented in Fig. 5. The curves are consistent with Michaelis–Menten kinetics (Eq. 2) at each pressure. The corresponding *K*_M_ and *k*_cat_ values are listed in Table 4. Figure 5 shows that increasing the pressure reduced the reaction rate. Correspondingly, the *k*_cat_ values decreased with increasing pressure (approximately threefold between 5 and 132 MPa; Table 4), but, remarkably, the values of *K*_M_ hardly changed. This shows that the studied range of pressures had no significant impact on the affinity of urease for the substrate.

#### Effect of pressure on* K*_M_

The effect of pressure on a chemical equilibrium at a constant temperature is to shift the existing equilibrium (*K*_0_) to a new position, wherein the equilibrium constant *K* changes as a function of pressure *p* as expressed by [32, 58]:

11 $$ K = K_{o} \;e^{{ - \tfrac{\Updelta V\,p}{RT}}} , $$

where Δ*V* is the reaction volume, i.e. the excess volume of products over reactants. The volume can be derived from the slope of the linear plot of ln*K* versus *p* (Eq. 11).

**Fig. 6:**
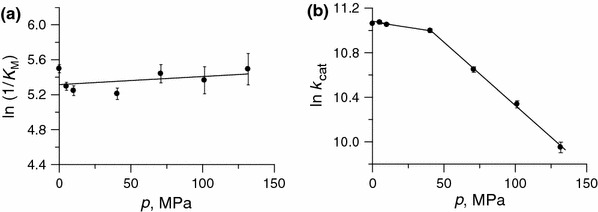
Effect of pressure in the range 0.1–132 MPa and at 25 °C on **a** the Michaelis constant *K*_M_ and **b** *k*_cat_ for the urease-catalyzed hydrolysis of urea. Points for 0.1 MPa were taken from the temperature-dependent measurements performed at ambient pressure at 25 °C (Table 1)

To determine the reaction volume for the binding step E + S ⇄ ES of the urease reaction (Δ*V*_b_), a plot of the inverse of the Michaelis constant 1/*K*_M_ versus *p* was drawn (Fig. 6a), and the binding volume Δ*V*_b_ was found to be −2 ± 2 mL/mol (Table 5). Although close to zero, this small negative value may suggest that the system shrinks slightly upon ES formation, due for instance to the expulsion of water from the active site into the bulk (Scheme 2). Most interestingly, however, the insignificant variance of *K*_M_ with pressure proves that pressures up to 132 MPa barely have an impact on the architecture of the active site of urease, and that the binding of urea to the site is practically unperturbed.

#### Effect of pressure on* k*_cat_

As predicted by the absolute rate theory, the dependence of the reaction rate constant *k* on pressure *p* is expressed by [32, 58]:

12 $$ k = k_{\text{o}} \;e^{{ - \tfrac{{\Updelta V^{ \ne } \,p}}{RT}}} , $$

where Δ*V* ^*≠*^ is the activation volume. In principle, the activation volume is the difference between the volume of reactants and their volume in the transition state of the reaction. For enzymatic reactions, Δ*V* ^*≠*^ refers to the catalytic step ES → (ES–EP)^*≠*^, and is determined experimentally from the slope of the linear plot of ln *k*_cat_ versus *p* (Eq. 12); however, as will be argued later, interpreting it may not be as simple as defining it.

In point of fact, consisting of contributions from both the catalytic and binding step, the overall activation volume Δ*V* ^*≠*^ has a value that depends on substrate concentration [32]. If the Michaelis–Menten mechanism is assumed for the enzyme reaction (Eq. 1), the initial reaction rate expressed by Eq. 2, when differentiated with respect to pressure, becomes:

13 $$ \Updelta V^{ \ne } = \Updelta V_{\text{cat}}^{ \ne } - \frac{{K_{\text{M}} }}{{K_{\text{M}} + S}}\Updelta V_{\text{b}} . $$

Equation 13 reveals that, upon decreasing the substrate concentration *S*, the binding contribution to Δ*V* ^*≠*^ increases up to the limit Δ*V* ^*≠*^ =  $\Updelta V_{\rm cat}^{ \ne }$ − Δ*V*_b_, whereas at the saturating substrate concentration the contribution of the binding volume becomes negligible and Δ*V* ^*≠*^ = $\Updelta V_{\rm cat}^{ \ne }$, which is the case analyzed here. Note that the overall Δ*V* ^*≠*^ can be obtained independently from the dependence of ln (*K*_M_/*k*_cat_) on *p* (Eq. 11 divided by Eq. 12, Table 5).

**Fig. 7:**
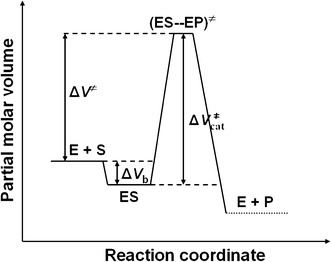
Schematic volume profile for the urease-catalyzed hydrolysis of urea

The plot of ln *k*_cat_ versus *p* for the studied urease–urea system is presented in Fig. 6b. The effect of pressure on *k*_cat_ was found to be biphasic. Though not quite typical, such behaviour of enzymatic systems is not infrequent [53, 61]. In the case of urease, the *k*_cat_ value of the enzyme decreased over the whole of the pressure range studied. The decrease up to 40 MPa was only to 94 % of the initial value, but the decrease was threefold upon going from 40 to 132 MPa. The resulting activation volume $\Updelta V_{\rm cat}^{ \ne }$ changed from 5 ± 1 mL/mol at *p* < 40 MPa to 28 ± 1 mL/mol at *p* > 40 MPa (Table 5). Compared to the binding volume Δ*V*_b_ obtained from *K*_M_, these results prove that the variation of enzyme activity with pressure is mainly due to the catalytic step of the reaction. In summary, we propose a volume profile for the urease-catalyzed hydrolysis of urea (Fig. 7), which schematically presents the volume changes that occur along the reaction coordinate, where $\Updelta V_{\rm cat}^{ \ne }$= Δ*V* ^*≠*^ + Δ*V*_b_.

Various pressure-induced changes in enzyme systems can be considered to be responsible for the nonlinear pressure dependence of enzyme kinetic parameters. These include, in addition to protein structural changes, compressibility changes and changes in the rate-determining step [53]. As argued earlier, urease retains its activity even in the dissociated form, and we also showed that the active site is not particularly perturbed by elevated pressures. Therefore, in order to account for the observed effect of pressure, we need to analyze the suggested reaction mechanisms in more detail.

It is clear from the analysis of the coordination spheres of the Ni(1) and Ni(2) centers in the active site of urease (Scheme 2) that the pentacoordinate Ni(1) is more electrophilic than the hexacoordinate Ni(2), which explains why urea first binds via the O-donor to Ni(1) before its amide group interacts with Ni(2). Furthermore, from the presence of the negatively charged coordinated ligands (Lys–NH–CO_2_, bridging OH, and O–Asp), it can be inferred that the p*K*_a_ of water–Ni(1) is considerably lower than that of water–Ni(2), which is due to the extra negative charge from O–Asp on Ni(2). However, more importantly, as it is bound to two Ni centers, the p*K*_a_ of the bridging OH group is expected to be lower than that of the OH bound to either Ni(1) or Ni(2). For that reason, the reaction path outlined in Scheme 2b seems more convincing to account for the catalytic hydrolysis, where the bridging hydroxo group supplies the proton required to initiate the process [5, 62]. In terms of the arguments outlined above, the experimentally observed p*K*_a_ values of 5.3 and 6.6 can be viewed as being related to the carboxyl and imidazole groups at the active site [16], while the p*K*_a_ value of 9.1 could be related to the deprotonation of the bridging OH group [16]. The latter step then accounts for the proton transfer following the nucleophilic attack of the bridging OH on the urea amide group to form NH_3_. The above conclusions are in keeping with the urease pH–activity profile with an optimum pH of ~7.5 [16].

Given the results of this work on the catalytic activity of urease, and those reported in the literature [5, 27, 62], we fully support the overall reaction mechanism put forward by Benini et al. [5], in which the bridging OH—not the terminal W2—is a nucleophile (Scheme 2b). This mechanism, proposed in [5, 62], is illustrated in Scheme 3 [62]. In the mechanism, in step A → B urea enters the active site when the flap is open, to bind to Ni(1) via the carbonyl O-donor, which involves the release of three water molecules. During step B → C, the flap closes and the urea NH_2_ coordinates to Ni(2). This is followed by nucleophilic attack by the bridging OH to produce the tetrahedral intermediate D. A proton transfer then occurs in step D → E to form C–NH_3_^+^, which is stabilized by the neutral imidazole of His323 from the active-site flap. Finally, C–N bond cleavage occurs to release NH_3_ and carbamate in the last step E → A, which is accompanied by flap opening and the uptake of four water molecules to yield A.

In general, it should be kept in mind that interpreting the activation volume is not always a straightforward task, because the experimentally obtained value could be the sum of three contributions: an intrinsic contribution that arises from structural volume changes in the molecules due to bond formation and bond scission processes; a solvational contribution resulting from the rearrangement of water molecules during the reaction, which is especially pronounced when charge and dipole changes occur in the reacting molecules; and a conformational contribution associated with changes in the conformation of the enzyme that accompany substrate binding and chemical steps. In terms of the observed pressure dependence of *k*_cat_, the binding of urea and the release of three water molecules in step A → B can be accompanied by a significant overall volume increase in the transition state. The flap closure process in step B → C, on the other hand, is expected to be accompanied by a significant volume decrease. The subsequent steps C → D → E, involving intramolecular nucleophilic attack and proton transfer, are not expected to be accompanied by significant volume changes. In the final step E → A, the volume increase associated with the release of NH_4_^+^ and NH_2_COO^−^ should mostly be canceled out by the volume decrease associated with the uptake of four water molecules, so this step is not expected to contribute meaningfully to the observed pressure effect. Thus, the significant decrease in *k*_cat_ with increasing pressure can mainly be ascribed to the first step of the catalytic cycle, which involves the release of three water molecules during the binding of urea to the active site. In general, deprotonation equilibria are characterized by negative reaction volumes due to an increase in electrostriction as a result of charge creation. Since the studied reaction is accelerated by the deprotonation of the carboxyl and imidazole groups in the lower pH range, their deprotonation is expected to be accompanied by a negative reaction volume, which could, in principle, account for the weaker effect of pressure observed in the low-pressure range (0.1–40 MPa), since the expected volume increase due to the reaction A → B will be partially canceled out by the negative reaction volume associated with the deprotonation process. However, at higher pressures, this contribution is expected to be canceled out by the effect of the selected buffer, since the latter is independent of pressure and will stabilize the pH of the solution to prevent further deprotonation of these groups at higher pressures in the range 40–132 MPa.

We therefore conclude from the above interpretation of the high-pressure kinetic results obtained in this study that the data corroborate the catalytic mechanism proposed by Benini et al. [5], which is outlined in Scheme 2b and described in more detail in Scheme 3 [62].
